# The Impact of COVID-19 Infection on Cognitive Function and the Implication for Rehabilitation: A Systematic Review and Meta-Analysis

**DOI:** 10.3390/ijerph19137748

**Published:** 2022-06-24

**Authors:** Sarah Houben, Bruno Bonnechère

**Affiliations:** 1 Scientific Direction Infectious Diseases in Humans, Sciensano, 1050 Brussels, Belgium; sarah.houben@sciensano.be; 2 REVAL Rehabilitation Research Center, Faculty of Rehabilitation Sciences, Hasselt University, 3590 Diepenbeek, Belgium; 3 Technology-Supported and Data-Driven Rehabilitation, Data Sciences Institute, Hasselt University, 3590 Diepenbeek, Belgium

**Keywords:** long-COVID, cognitive disorders, rehabilitation

## Abstract

There is mounting evidence that patients with severe COVID-19 disease may have symptoms that continue beyond the acute phase, extending into the early chronic phase. This prolonged COVID-19 pathology is often referred to as ‘Long COVID’. Simultaneously, case investigations have shown that COVID-19 individuals might have a variety of neurological problems. The accurate and accessible assessment of cognitive function in patients post-COVID-19 infection is thus of increasingly high importance for both public and individual health. Little is known about the influence of COVID-19 on the general cognitive levels but more importantly, at sub-functions level. Therefore, we first aim to summarize the current level of evidence supporting the negative impact of COVID-19 infection on cognitive functions. Twenty-seven studies were included in the systematic review representing a total of 94,103 participants (90,317 COVID-19 patients and 3786 healthy controls). We then performed a meta-analysis summarizing the results of five studies (959 participants, 513 patients) to quantify the impact of COVID-19 on cognitive functions. The overall effect, expressed in standardized mean differences, is −0.41 [95%CI −0.55; −0.27]. To prevent disability, we finally discuss the different approaches available in rehabilitation to help these patients and avoid long-term complications.

## 1. Introduction

In December 2019, the first cases of the new severe acute respiratory syndrome coronavirus 2 (SARS-CoV-2) were reported in Wuhan, China [1]. Coronavirus disease 2019 (COVID-19) is caused by coronavirus 2 causing severe acute respiratory syndrome (SARS-CoV-2). COVID-19 has rapidly spread all over the world despite important efforts (i.e., lockdown, quarantine, social distancing) made to try to contain it [2]. On 17th May the total number of detected cases was more than 522 million and the total number of deaths was 6,267,500 [3]. The majority of individuals infected with COVID-19 experience mild-to-moderate illness, while approximately 10–15% develop severe illness and 5% become critically ill [4]. Depending on the severity of symptoms, the average duration of recovery from COVID-19 is two to three weeks [5].

Even though pulmonary impairments are the most prevalent manifestation of COVID-19, extrapulmonary manifestations are abundant [6], and there are increasing pieces of evidence in favour of an extra-respiratory spreading from the coronaviruses. For the large majority of people, the recovery after COVID-19 infection is complete within 12 weeks. However, there will be a large number of recovered COVID-19 patients who may experience a variety of long-term health effects. Even though the multi-organ manifestations of COVID-19 are now well-documented, the potential long-term consequences of these manifestations remain unknown. People with COVID-19 might have sustained post-infection sequelae. Known by a variety of names, including long COVID or long-haul COVID, and listed in the ICD-10 classification as post-COVID-19 condition since September 2020, this occurrence is variable in its expression and impact [7]. Post-COVID-19 condition occurs in individuals with a history of probable or confirmed SARS-CoV-2 infection, usually 3 months from the onset, with symptoms that last for at least 2 months and cannot be explained by an alternative diagnosis. Common symptoms include, but are not limited to, fatigue, shortness of breath, and cognitive dysfunction, and generally have an impact on everyday functioning. Symptoms might be new-onset following initial recovery from an acute COVID-19 episode or persist from the initial illness. Symptoms might also fluctuate or relapse over time [7]. It is estimated that 1 out of 10 patients may have symptoms lasting 12 weeks or more [8]. In this context, the accurate and accessible assessment of cognitive functions in patients post-COVID-19 infection is thus of increasingly high importance for both public and individual health. Usually, cognition is divided into several sub-functions such as attention, memory, language, and visuospatial abilities [9,10]. These sub-functions are for example each impacted differently by the process of aging [11] but little is known about the influence of COVID-19 on the general cognitive levels and more importantly at the sub-functions level. ‘Post COVID’ clinics have been created in various countries, especially in Europe, for the management of people affected by long COVID syndrome. Guidelines have been written to help clinicians. An important role in the management of long COVID patients is played by the general practitioner, directly or indirectly linked to post-COVID hospital clinics. The extreme heterogeneity of clinical presentation needs a patient-tailored, multidisciplinary approach; note that only very limited information was available concerning the rehabilitation of these patients [12].

Therefore, this study has two main objectives. We first aim to summarise the current level of evidence supporting the negative impact of COVID-19 infection on cognitive functions. Then we present and discuss the different potential interventions available in rehabilitation to try to decrease the risk of cognitive disorders after COVID-19 infection and restore optimal cognitive functions in patients presenting long COVID symptoms.

## 2. Methods

The protocol of the present study was registered in the International Prospective Register of Systematic Reviews PROSPERO (registration number CRD42022303425).

### 2.1. Search Strategy

Records were searched on three databases (Pubmed, Biber, and Scopus) to identify eligible studies published before April 2022.

The search strategy was built around the relationship between COVID-19 and cognitive functions. The search terms included a combination of the following MeSH terms and free words: COVID-19: (COVID* OR SARS-CoV-2); COGNITION: (“cognit*” OR “memory” OR “attent*” OR “intellect” OR “executive funct*” OR “recognit*” OR “IQ” OR “problem solving” OR “psychomotor speed” OR “mental flexib*” OR “choice react*” OR “emotional bias” OR “planning” OR “response inhibition”). References from selected papers and from other relevant articles were screened for potential additional studies in accordance with the snowball principle. The search was limited to journal articles published in English.

### 2.2. Eligibility Criteria

A PEO approach was used as inclusion and exclusion criteria, which were assessed by the study team [13].

- **Population**: Healthy adults (without pre-existing conditions) with COVID-19 diagnosed using PCR. Studies with patients suffering from neuropsychiatric disorders before the infection were therefore not included in this analysis.
- **Exposure**: COVID-19 infection.
- **Outcome**: Any outcomes related to cognitive disorders, loss of cognitive functions, and/or cognitive fatigue.

**Figure 1:**
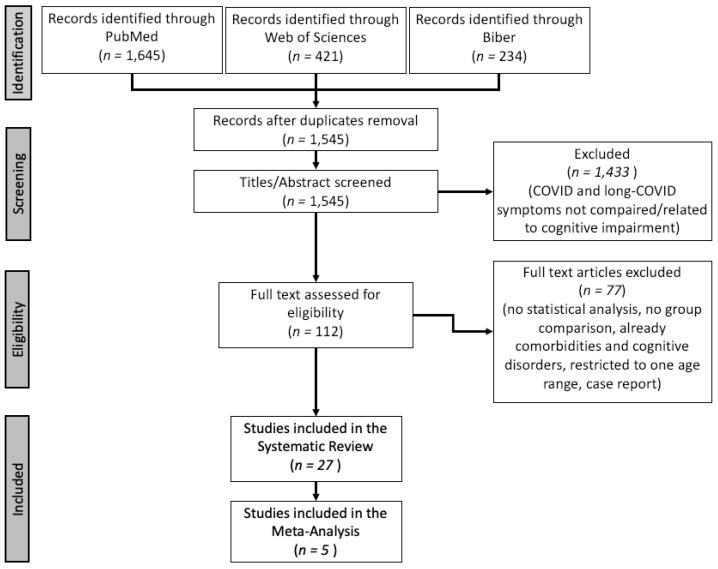
Flowchart of study selection.

A flow diagram of the study selection with the screened articles and the selection process is presented in Figure 1.

### 2.3. Data Extraction

The following information was extracted from the included studies: characteristics of the patients (age, sex ratio, education level), main outcomes, cognitive (sub)-functions assessed, and period of recruitment.

### 2.4. Quality Assessment

The critical appraisal of the methodology was based on the Newcastle–Ottawa Scale (NOS) [14]. The following thresholds were used to convert the NOS to the Agency for Healthcare Research and Quality (AHQR) standards [15]: Good quality: 3 or 4 stars in the selection domain and 1 or 2 stars in the comparability domain and 2 or 3 stars in outcome/exposure domain. Fair quality: 2 stars in the selection domain and 1 or 2 stars in the comparability domain and 2 or 3 stars in outcome/exposure domain Poor quality: 0 or 1 stars in the selection domain or 0 stars in the comparability domain or 0 or 1 stars in outcome/exposure domain.

### 2.5. Statistical Analysis

For studies assessing the efficacy of a rehabilitation program, we performed a meta-analysis. The measure of treatment effect was the standardized mean difference effect size (standardized mean difference (SMD)), defined as the between-group difference in mean values divided by the pooled SD computed using the Hedge’s g method. If different tests were used to assess the same cognitive sub-functions in the same study, the different results were pooled to have one unique SMD as recommended by Cochrane’s group [16]. A positive SMD implies an increased risk of lower cognitive function compared to the control. We assessed the heterogeneity in stratified analyses by type of cognitive sub-functions. We calculated the variance estimate tau^2^ as a measure of between-trial heterogeneity. We prespecified a tau^2^ of 0.0 to represent no heterogeneity, 0.0–0.2 to represent low heterogeneity, 0.2–0.4 to represent moderate heterogeneity, and above 0.4 to represent high heterogeneity between trials [17]. To deal with high or moderate heterogeneity we used random-effect models and presented forest plots for the different cognitive functions. We checked for publication bias using a funnel plot [18] and Egger’s test for the intercept was applied to check the asymmetry [19].

### 2.6. Ethical Approval

This review was reported following the Preferred Reporting Items for Systematic Reviews and Meta-Analyses (PRISMA) recommendations [20]. For the present study, no ethics committee approval was necessary.

## 3. Results

### 3.1. Search Results

Twenty-seven studies were finally included in the systematic review. The PRISMA flowchart of the study selection is presented in Figure 1.

### 3.2. Characteristics of the Participants

Participants numbering 94,103 were included in this review: 90,317 COVID-19 patients and 3786 control. The mean age was 53.8 (10.4) years old and the level of education 12.6 (2.7) years. There were more females than males (52% of the COVID-19 patients were females, 54% in the control group). Most of the studies were performed during the first wave of the pandemic. Characteristics of the included studies and the patients are presented in Table 1. 

### 3.3. Systematic Review

First, concerning the quality of the papers, most of them (*n =* 20, 74%) were ranked as of good quality according to the AHQR standards using the NOS. The seven other studies (26%) were ranked as of fair quality.

The main results of the included individual studies are presented in Table 2.

First, concerning the methodology, we can see that the most frequent test to assess the cognitive function of COVID-19 patients is the Montreal Cognitive Assessment (MoCA). MoCA was used in 10 out of the 27 studies (37%) [23,24,27,30,36,38,39,43,44,45], followed by the Mini-Mental State Examination (MMSE) in four studies (15%) [23,32,43,44] and the Trail Making Test (TMT) also in four studies [22,25,28,34]. Most of the other tests and scales were only used in one or two individual studies.

Given the nature of this pandemic, it was needed to perform the evaluation in another way than in person during clinical testing. The MoCA was the most used test and has been found to be reliable to detect mild cognitive deficits and is available and validated in nearly 100 languages [48]. This test was efficient to highlight differences between control and COVID-19 patients in various studies and, contrary to MMSE, MoCA seems to be able to bring out sub-clinical defects and more clearly discriminate differences between ability levels [43].

All the studies reported deficits in cognitive functions after COVID-19. However, the magnitude of the effects varied quite strongly in the various studies. Several factors could explain these differences.

The first one is the age of the patients. In two different studies, the authors showed that the cognitive deficits were correlated with the age of the patients: older patients tended to have more severe deficits compared to younger ones [23,27]. However, other authors found that even younger patients also experience mild cognitive deficits after COVID-19 recovery, regardless whether they were affected by mild or moderate symptoms [21]. These results are confirmed by other studies indicating that there was also a significant rate of cognitive impairment in young adults [21,31,39]. Davis et al., 2021, point out that cognitive dysfunctions affected 88% of their participants, independently of their age [26].

A second important point that could modify the impact of COVID-19 on cognitive functions is the initial cognitive status of the participants (i.e., the influence of previous cognitive deficits). In a study evaluating patients with cognitive deficits before the disease, authors showed that there were no significant differences between patients and controls [43]. Another study identified cognitive disorders like Alzheimer’s disease and dementia as risk factors for hospital admission after the development of the COVID-19 disease, but not a more important decrease in cognitive functions [42]. Poletti et al., 2021, evaluated the cognitive performances of COVID-19 patients already suffering from major depression [37]. In the two COVID-19 recovery groups, patients suffering from depression had lower scores in cognitive functions compared to healthy controls.

Finally, an important question is to determine whether or not the severity of the infection (COVID-19) influences cognitive impairment. In the study by Van den Borst et al., 2020, 124 patients with different stages of COVID-19 (mild, moderate, severe, and critical) were included [40]. They observed that patients with mild symptoms were more likely to suffer from fatigue than patients with more severe stages but for the cognitive deficits, the severity of the disease was not correlated. Another study showed that there was an association between cognitive sequelae and the severity of lung affection and restricted cerebral oxygen delivery [34]. Mendez et al., 2021 showed that hospitalized COVID-19 patients had a considerable rate of neurocognitive impairment: 58.7% of the patients with moderate or severe COVID-19 pathology presented a moderate neurocognitive deficit and 18.4% presented a severe one [33]. Hamshire et al., 2021 showed that there is a significant decrease in cognitive performance in patients, depending on their level of medical assistance following their SARS-CoV-2 infection [29]. However, in most of the other studies, the stage of COVID-19 infection was not correlated with the appearance of cognitive deficits and their severity [21,35]. Interestingly in another study, authors showed that the patients do not present neurological deficits or cognitive impairments, but seemed to present severe emotional disorders compared to the control group, which could explain different levels of motivation and thus, cognitive functions [32].

A positive point is that, despite important differences in study duration and follow-up, it seems that 6 months after COVID-19 recovery, an improvement in cognitive functions was observed [27,36,44], although differences persist with the initial value.

To further investigate the impact of COVID-19 on cognitive functions some authors performed complementary analysis using neurophysiological measurements. Some authors tested patients for neurophysiological disorders with 18-FDG-PET [28,30], CSF analysis [30], MRI [26,27,30], EEG [27], blood biomarkers [22,42], or complete neurological examination including cranial nerve exam, strength, reflexes, sensory and coordination functions, when patients showed cognitive deficits after completion of the evaluation tests [32]. Some COVID-19 patients showed cortical hypometabolism (highlighted by 18-FDG-PET scan) [30]. CSF analysis did not reveal any abnormalities and did not reveal the presence of SARS-CoV-2 after RT-PCR [30]. Interestingly, it seems that there is no visible manifestation of COVID-19 visible in MRI [26,45]. However, in another study, four participants (out of 27) presented micro embolic subacute infarcts but did not present any other structural changes [30]. Another study reported EEG abnormalities but only in two out of 50 individuals [27]. In some cases, post-mortem analyses were performed: pronounced microgliosis, with microglial nodules, and astrogliosis were found in patients who died after COVID-19 infection [30,49]. These examples are rather anecdotic and in the vast majority of the cases, there are no visible modifications in the brain.

### 3.4. Meta-Analysis

**Figure 2:**
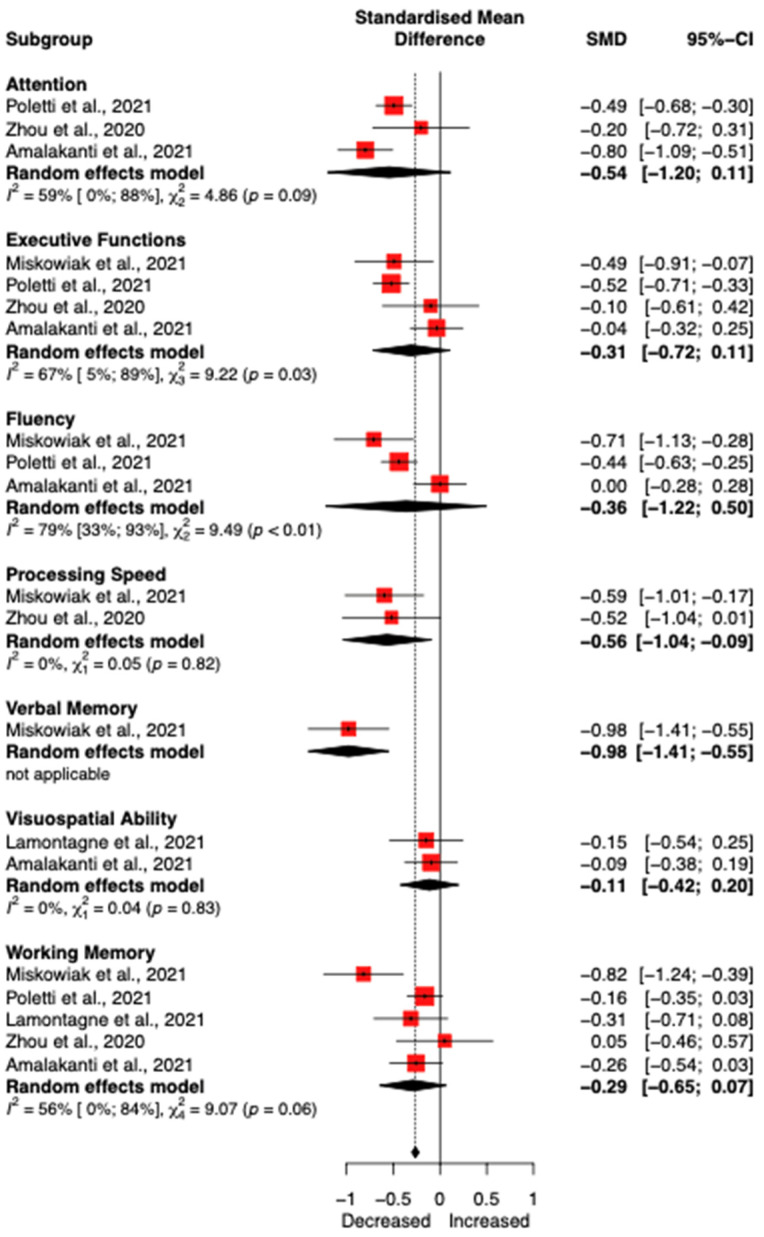
Stratified meta-analysis according to cognitive sub-functions. Results are indicated with 95% confidence intervals. Negative Standardized Mean Difference (SMD) indicates a decrease in cognitive functions in COVID-19 patients compared to healthy individuals [22,24,31,34,37].

Out of the 27 studies included in the systematic review, five (959 participants, 513 patients) were included in the meta-analysis to quantify the impact of COVID-19 on cognitive (sub)functions [22,24,31,34,37]. First, we assessed the overall effect of COVID-19 on cognitive functions. Out of the five included in the study—including all the different tests—long COVID-19 patients had, on average, a decrease of −0.41 [95% CI −0.55; −0.27] (using fixed effect model due to low heterogeneity (Tau2 = 0.0047, *p* = 0.32)). Next, we analysed the differences at the sub-cognitive function levels. Statistically significant differences were found between the different cognitive functions (*p* < 0.001), but the results of this analysis should be interpreted carefully due to the limited number of studies available for the different cognitive functions. The forest plot is presented in Figure 2.

## 4. Discussion

The aim of this review was first to quantify the level of cognitive disorders in patients with confirmed COVID-19 and more specifically during long-COVID.

### 4.1. Main Findings

By analysing the results of the different studies, there is clear evidence that people infected by SARS-CoV-2 show significant cognitive disorders (mean SMD −0.41 [95% CI −0.55; −0.27]), independently from the pathology stage or patients’ age. In addition, there is not a clear link between the severity of the infection and the degree of neurocognitive deficit. Before discussing the effect of rehabilitation, it is interesting to discuss other potential factors that could decrease the importance of the observed cognitive impairment in these patients.

The first potential aspect is vaccination. Most of the studies recruited the participants during the first wave, in 2020. Since fifty percent of the worldwide population was fully vaccinated (two doses of vaccine) at the beginning of January 2022, the vast majority of participants contracted COVID-19 before receiving any vaccination dose and it is therefore difficult to assess this point. To the authors’ best knowledge there is currently no study assessing the link between vaccination and a decrease in cognitive functions. It would be interesting to see new studies on long-COVID, among a fully vaccinated population. Nevertheless, it seems that vaccinated people with breakthrough infection were partially at lower risk of death and post-acute sequelae than people with a SARS-CoV-2 infection without prior vaccination, but cognition was not evaluated in this study [50]. Another study showed that vaccination may reduce the burden of long-COVID and this is already the case after one dose of vaccine [51]. Similar results were found in another study which concluded that people, especially older than 60 were more likely to be asymptomatic if they were infected by SARS-CoV-2 after being fully vaccinated [52]. So, if the pathology is less aggressive and the symptoms are reduced in vaccinated people, we could assume that the cognitive impairment in these infected people would be less important.

Nevertheless, according to OpenVAERS, 1,301,354 adverse events were reported after COVID-19 vaccine administrations and among that, there are more than 163,000 hospitalizations (data from the 20th of June 2022) [53]. Different vaccines were tested during this period and some of them were recalled or restricted due to side effect issues. We can easily imagine that cognitive impairments or reduced quality of life were among these adverse events and therefore we cannot exclude a possible role of the vaccination in the observed neurological deficits. Another aspect to analyse is the age of the included participants. Here, for our systematic review, we chose adults as inclusion criteria, however, long-COVID pathology has been also reported in children and adolescents, but only a few studies have analysed long-COVID in the paediatric population [54,55,56]. It is however important to note that there are very limited cases of children with severe SARS-CoV-2 infection. Children and young adults affected by COVID-19 tend to be less sick than older adults and therefore we can assume that they presented a reduced risk of potential neurological disorders. It is also not clear whether the cognitive disorders that could be observed were due to the SARS-CoV-2 infection or to the pandemic situation where restrictions, isolation, and online teaching for example have been important stress factors that could also directly negatively impact cognitive functions. Studies in this population is surely necessary because they are still in a crucial phase of brain development.

Although some groups began to explore the neurophysiology [22,31] and the neuropathology [30,49] behind these cognitive impairments, the mechanism of action between SARS-CoV-2 infection and cognitive disorders is far from being understood. Current evidence suggests a highly multifactorial component: direct infection by SARS-CoV-2, the consequence of prolonged-time spent in intensive care units, persistent inflammation, brain hypoxia, ventilation mechanisms used, drugs, prior cognitive troubles, and peripheral organ dysfunction. The combination of these factors could lead to the so-called long-COVID statement. The uncontrolled inflammatory response, also named the cytokine storm may contribute to the severity of the disease. This increased level of inflammatory cytokines and chemokines was previously also observed during infection with other severe coronaviruses. High levels of IL-6, IL-8, and TNF-α were found in COVID-19 patients’ serum [22,57].

Some suggest that the sustained inflammatory response could contribute to psychiatric sequelae, such as cognitive impairment, after COVID-19 [31]. This persistence of inflammation was already correlated with depression [58] and can lead to a disruption of the blood–brain barrier (BBB), also resulting in neuronal and glial cells damages [59]. BBB permeability will permit cytokines like IL-6 to enter the brain giving rise to depression-like behaviours [60]. The disruption of the BBB can also directly permit SARS-CoV-2 to reach the central nervous system, in addition to the other pathway that would be the retrograde transport via the olfactory sensory neurons [61].

However, on the other hand, this cytokine storm is only observable in the most severe cases, and we have seen that these cognitive impairments affect patients who have had either mild or severe forms of COVID-19 [50]. Therefore, this mechanism alone could not (fully) explain the neuropsychiatric deficits.

As with other coronaviruses, SARS-CoV-2 shows a neurotropism. The virus could enter into neurons and glial cells with the SPIKE protein, which binds to ACE2 receptors (angiotensin-converting enzyme 2) [62], which would result in neuronal death, and then, cause cognitive deficits [59]. As adult neurogenesis is not yet clearly demonstrated, this neuronal loss would be irreversible and could lead to an acceleration decline of brain functions, causing the typical symptoms observed in pathologies such as Alzheimer’s disease, and Parkinson’s, namely memory loss, learning deficits, and motor problems for example.

### 4.2. Limitations of the Systematic Review

The findings of this study have to be seen in the light of some limitations. First, as seen in Table 2, there is a huge variety of tests and scales used to assess cognitive disorders making the comparison between studies difficult. It is important to note that most of the studies have used the MoCA but, although this test is convenient and easy to administer, it may not be the most sensitive in detecting small modifications of cognitive functions [63]. Another limitation is the low number of studies included in the meta-analysis; this is mainly explained by the fact that most of the studies are trying to compare the potential effect of the severity of the disease on the cognitive symptoms rather than comparing the results with healthy controls. We could have expanded the scope of this review and also included studies assessing the quality of life after COVID [64], since the decrease in cognitive function or the perception of increased cognitive fatigue both have a direct impact on quality of life [65]. However, due to the fact that we had already been using quite heterogeneous tests and scales, we decided to restrict this analysis purely to cognitive functions. Nevertheless, a recent review on the impact of COVID on quality of life shows that the most common problems that affected patients’ quality of life at 6 and 12 months are fatigue or muscle weakness (Pooled Prevalence (PP) 6–12 m = 54.21%, PP ≥ 12 m = 34.22%), mild dyspnea (Modified Medical Research Council Dyspnea Scale, PP 6–12 m = 74.60%, PP ≥ 12 m = 80.64%), anxiety and depression (PP 6–12 m = 33.49%, PP ≥ 12 m = 35.40%), pain or discomfort (PP 6–12 m = 33.26%, PP ≥ 12 m = 35.31%) and difficulty concentrating (PP 6–12 m = 22.47%, PP ≥ 12 m = 29.47%), highlighting the importance of cognitive impairment on quality of life [66].

Despite these limitations, we found an important cognitive burden associated with (long)-COVID. Most of the included studies highlighted the importance of rehabilitation in long-COVID patients, but also the need for a rapid assessment of these patients (i.e., associated risk factors, prior cognitive deficits, etc.) at the early phase of the disease to potentially identified the patients who were more likely to benefit from rehabilitation [23,25,35,36,44,47,67]. Therefore, in the next part of the discussion, we focus on different potential interventions available in rehabilitation to improve this condition.

### 4.3. Rehabilitation Strategies

Different rehabilitation strategies have been proposed to improve the functions and the quality of life of patients suffering from COVID-19 infection both in the acute [68] and the chronic phase [69]. In the acute phase, rehabilitation seemed to improve dyspnoea, anxiety, and kinesiophobia. Results on pulmonary function were inconsistent, while improvements were detected in muscle strength, walking capacity, sit-to-stand performance, and quality of life, no information was available for cognitive functions [68].

Of course, most of the interventions, and therefore the current level of evidence, are focusing on pulmonary rehabilitation [70] and physical activity [71,72]. Significant differences were also found in quality-of-life related outcomes for both short and long term.

A new model of care has emerged, utilizing information and communication technologies to ensure the continuation of these services. Health services delivered via digital means are referred to as “telehealth”, “eHealth”, or “mHealth” [73]; about physiotherapy, the term “telerehabilitation” has been widely used in the literature to describe rehabilitation services delivered via mHealth [74]. Telerehabilitation can be provided through a variety of digital channels, including synchronous audio and/or video calls, as well as asynchronous channels such as recorded videos, text messages, emails, and links to educational materials [75]. Three randomized control trials (RCT) have been recently published on the use of telehealth in the management of COVID-19 patients.

In the acute phase of COVID-19, it has been shown in a large RCT that delivering breathing exercises via telerehabilitation was a promising, safe, and effective strategy for improving physical performance, dyspnoea, and perceived effort [76]. Patients performed breathing exercises at home once per day for one week, while a physiotherapist reinforced the program via videoconference; patients also received a daily text message to increase adherence.

In another study, the authors examined the effects of a 6-week unsupervised home-based exercise program consisting of breathing, aerobic, and lower limb muscle strength exercises delivered to COVID-19 patients via smartphone and remotely monitored by heart telemetry. At week 6 (post-treatment) and week 28 (follow-up), the intervention was superior in terms of exercise capacity, lower limb muscle strength, and quality of life [77].

In a last RCT the authors compared the efficacy of two different exercise-based programs (strengthening and breathing exercises) delivered via telerehabilitation in COVID-19 patients [78]. After the 14-day intervention, statistically significant differences were observed between the two intervention groups and the control group in all variables (fatigue, dyspnoea, perceived effort, and physical condition), with the breathing exercises group showing the greatest improvements in dyspnoea and aerobic capacity.

The three examples show that telerehabilitation proved to be an effective, safe, and feasible modality to facilitate the recovery of these patients, but it must be noted that specific outcomes related to cognition were never investigated in the above-mentioned studies. However, based on previous works and evidence, mainly studies on aging population, we can assume that physical exercises and an increased physical activity level will not only induce an increase in motor outcomes but will also improve cognition. It has indeed been shown that older people who are regularly engaged in exercise are more likely to maintain their cognitive functions compared to those who are physically inactive [79]: as a matter of fact, exercise has been shown to be a highly effective therapeutic strategy for age-related progressive neurodegenerative disorders, including dementia [80], with greater levels of physical activity seemingly protective against the onset of dementia in individuals who are healthy at baseline. In addition, physical activity yields significant improvements in cognition in individuals with dementia and mild cognitive impairment [81,82,83]. Interestingly a recent meta-analysis of randomized controlled trials has shown that combining cognitive intervention and physical exercise results in superior benefits over either intervention alone on global cognition, memory, executive function, and attention in older adults with mild cognitive impairment [84].

### 4.4. Implications for the Rehabilitation

The COVID-19 pandemic has drastically changed our lives. During the different peaks of the crisis, the continuity of care can no longer be guaranteed [85]. Therefore, rehabilitation services were forced to modify and adapt the way they provide and deliver services [86]. These measures were proposed and adopted in a large number of countries; the proposed changes included the following: A multidisciplinary team should administer early mobilization, respiratory, outpatient, and long-term care rehabilitation interventions to critically ill SARS-CoV-2 patients. Home- and community-based rehabilitation can be provided through various methods, such as telerehabilitation and direct care. COVID-19 transmission prevention and protection measures are required for all patients receiving rehabilitation care [87].

The COVID-19 pandemic has accelerated the development and implementation of telehealth, with the number of healthcare interventions delivered via digital devices increasing exponentially, also due to the widespread availability of mobile technology. This may open up new perspectives and opportunities in the healthcare industry, as previous research has shown that telehealth is well-received by patients, leading to greater adherence [88,89] and patient satisfaction [90,91]. So far, we have seen that there is currently, to the authors’ best knowledge, no study that has been specifically focusing on the rehabilitation of cognitive fatigue and disorders in COVID-19 patients. However, there is currently a growing body of evidence supporting the use of mHealth and brain training games or apps to train and challenge the brain in different ways. Recent systematic reviews and meta-analyses reported cognitive improvement after intervention using cognitive mobile games in various conditions such as healthy aging [92], mild cognitive impairment [93], stroke [94], Parkinson’s disease [95], and multiple sclerosis [96].

Technology and social media-based interventions appear to be promising techniques for promoting health and well-being and are the only effective methods for delivering an intervention during a pandemic situation [97]. However, there also appears a need for the development of guidelines for social media usage to prevent probable hazards and fake news.

However, a few issues must be resolved before these solutions can be implemented in daily practice. First, and likely most important, is the acceptance of mHealth applications as rehabilitation interventions. Not only has the COVID-19 pandemic disrupted healthcare systems, but it has also accelerated the development, implementation, and recognition of mHealth in clinical settings [98]. Notably, the majority of measures taken during the crisis may be temporary, and it is hoped that efforts will continue in this direction once the crisis has passed. For instance, it will be necessary to revise the nomenclature of interventions, as mobile solutions are currently placed in the same categories as pharmaceuticals, posing validation and reimbursement challenges [99]. A further limitation is that the majority of analysed mHealth is currently being developed as part of research projects and is therefore not readily available to patients. This brings us to the second major current limitation, which is the lack of social security reimbursement. The organization and participation of healthcare systems in the revalidation process varies by country, so we will not discuss reimbursement in detail here. However, we know that the two most significant barriers to the implementation of telemedicine and telehealth for patients, regardless of their pathologies or specialties, are financial concerns and a lack of knowledge and experience with the use of (new) technology [100,101]. Most patients are familiar with smartphones, apps, and mobile technology, so familiarity with the technology should not be an issue for the majority of patients [102], whereas this can be a significant barrier for other diseases or patient groups (e.g., older adults with dementia) [103]. Efforts must also be directed toward the education of healthcare professionals, as they must be trained in the technology and know its limitations in order to encourage patients to utilize it.

## 5. Conclusions

The COVID-19 crisis has profoundly altered the organization of our society and challenged the different health care systems. While revalidation services have been greatly impacted during the different waves (acute management of patients), rehabilitation specialists are now faced with the challenge of managing long-term complications. Among these complications, we have shown in this review important complaints in cognitive functions. Even if most of these disorders diminish with time, on average 6 months after the first infection, it is important to develop strategies to improve the situation. There is currently little work that has been done focusing on the rehabilitation of cognitive functions, but the current evidence suggests that the best option would be a combination of physical rehabilitation exercises combined with cognitive training. The latter can be carried out using computerized solutions. In the future, it is important to think about the best way to integrate cognitive stimulations within physical rehabilitation since cognitive disorders are frequently associated with many pathologies requiring rehabilitation, not only COVID-19 as we have seen in this paper, but also for example stroke, multiple sclerosis, and Parkinson’s disease.

## Figures and Tables

**Table 1:** Main characteristics of the included studies and socio-demographic characteristics of the patients. Age results are presented as mean (SD) or median [p25–p75] according to the distribution.

Study	Country	Recruitment Period	Evaluation Period	Patients			Control		
				N [% female]	Age	Education	N [% female]	Age	Education
Woo et al., 2020 [21]	Germany	July 2020	3 months of follow-up	18 [55%]	42.2 (14.3)	>12	10 [40%]	38.4 (14.4)	>12
Zhou et al., 2020 [22]	China	Uns.	Uns.	29 [38%]	47.0 (10.5)	12.6 (2.8)	29 [59%]	42.5 (6.9)	12.4 (3.1)
Alemanno et al., 2021 [23]	Italy	March to June 2020	Follow-up: one month after home-discharge	87 [29%]	67.2 (12.9)	Uns.	/	/	/
Amalakanti et al., 2021 [24]	India	June and July 2020	Uns.	93 [52%]	36.2 (11.7)	Uns.	102 [55%]	35.6 (9.8)	Uns.
Becker et al., 2021 [25]	USA	April 2020 to May 2021	Uns.	740 [63%]	49.0 (14.2)	103 less than 12 years	/	/	/
Davis et al., 2021 [26]	56 different countries	September to November 2020	Follow-up: up to 7 months	3762 [79%]	18–80 years old	Uns.	/	/	/
Del Brutto et al., 2021 [27]	Ecuador	March to May 2020	Follow-up: up to 6 months	50 [63%]	62.7 (11.9)	Uns.	28 [63%]	62.7 (11.9)	Uns.
Dressing et al., 2021 [28]	Germany	June 2020 to January 2021	202.3 ± 57.5 days after first positive COVID-19-PCR	31 [64%]	54.0 (2.1)	Uns.	/	/	/
Hampshire et al., 2021 [29]	UK (75,910) and other (5427)	January 2020 to December 2020	Uns.	81,337 [55%]	46.7 (15.7)	*	/	/	/
Hosp et al., 2021 [30]	Germany	April to May 2020	Uns.	29 [38%]	65.2 (14.4)	13.2 (3.0)	/	/	/
Lamontagne et al., 2021 [31]	USA & Canada	January 2020 to March 2021	Uns.	50 [29%]	30.8 (9.9)	16.1 (2.9)	50 [35%]	29.1 (9.9)	15.5 (2.9)
Mattioli et al., 2021 [32]	Italy	February 2020	Follow-up: 4 months	120 [75%]	47.8 [26–65]	16 [8–18]	30 [73%]	45.7 [23–62]	18 [8–18]
Méndez et al., 2021 [33]	Spain	March to April 2020	1 year after hospital discharge	171 [42%]	58.0 [50–68]	11 [8–16]	/	/	/
Miskowiak et al., 2021 [34]	Denmark	March to June 2020	3–4 months and 12 months after discharge	29 [41%]	56.2 (10.6)	14.3 (3.9)	100 [59%]	56.0 (6.9)	14.3 (3.0)
Norrefalk et al., 2021 [35]	Sweden	Uns.	Follow-up: 6 months	100 [82%]	44.5 (10.6)	<9 years (1), 10–12 years (31), >12 years (61), other (7)	/	/	/
Patel et al., 2021 [36]	USA	March to August 2020	Uns.	77 [36%]	61.0 (16.6)	Uns.	/	/	/
Poletti et al., 2021 [37]	Italy	May 2020 to February 2021	Follow-up: 1–3 and 6 months	312 [62%]	52.6 (8.8)	Uns.	165 [44%]	50.5 (9.2)	Uns.
Rousseau et al., 2021 [38]	Belgium	March to July 2020	Follow-up: 3 months	32 [28%]	62 [49–68]	Uns.	/	/	/
Solaro et al., 2021 [39]	Italy	November 2020 to March 2021	Uns.	32 [41%]	53.7 (4.8)	Uns.	/	/	/
Van den Borst et al., 2021 [40]	Netherlands	April to July 2020	Follow-up: 3 months	124 [40%]	59.0 (14.0)	Low (30), Middle (34), High (60)	/	/	/
Vyas et al., 2021 [41]	India	April to August 2020	Uns.	300 [48%]	15–70 years old	Uns.	/	/	/
Zhou et al., 2021 [42]	China	Uns.	Uns.	1091 [47%]	57.1 (9.2)	Uns.	2793 [52%]	57.7 (8.6)	Uns.
Aiello et al., 2022 [43]	Italy	May 2020 to May 2021	Uns.	45 [89%]	63.3 (11.4)	11.0 (3.9)	/	/	/
Bonizzato et al., 2022 [44]	Italy	Uns.	Follow-up: at discharge and after 3 months	12 [42%]	71.3 (10.1)	7.2 (3.3)	/	/	/
Del Brutto et al., 2022 [45]	Ecuador	May to June 2020	Uns.	50 [63%]	62.7 (11.9)	Uns.	28 [63%]	62.6 (11.8)	Uns.
Liu et al., 2022 [46]	China	February to April 2020	Uns.	1438 [52%]	69 [66–74]	12 [9–12]	438 [49%]	67 [66–74]	12 [9–12]
Tabacof et al., 2022 [47]	USA	March 2020 to March 2021	Uns.	156 [69%]	44 [13–79]	Uns.	/	/	/

* 94 (no schooling), 1553 (primary school), 28,827 (secondary school), 47,486 (university degree), 3294 (PhD), 83 (Unknow). Uns. = Unspecified.

**Table 2:** Description of the tests used to assess the cognitive function and main results of the included studies.

Study	Assessment Methods	Main Results	Quality *
Woo et al., 2020 [21]	Modified Telephone Interview for Cognitive Status (TICS-M)	Sustained sub-clinical cognitive impairments might be a common complication after recovery from COVID-19 in young adults.	Fair
Zhou et al., 2020 [22]	Trail Making Test (TMT), Sign Coding Test (SCT), Continuous Performance Test (CPT), and Digital Span Test (DST)	The study indicated a potential cognitive dysfunction in patients with COVID-19. Sustained attention is linked with the inflammatory level as indicated by CRP.	Fair
Alemanno et al., 2021 [23]	MoCA and MMSE	80% (out of 87 patients) showed neuropsychological impairments and 40% showed mild-to-moderate depression. They partly recovered at one-month follow-up and 43% had post-traumatic stress disorder signs. Those with severe functional deficits showed important cognitive and emotional deficits which might have been influenced by the choice of ventilatory therapy but seem to be age-related.	Good
Amalakanti et al., 2021 [24]	MoCA	Even otherwise asymptomatic COVID-19, patients have cognitive impairments, suggesting the need for a detailed psychometric assessment, especially in the elderly population.	Good
Becker et al., 2021 [25]	Number Span forward (attention) and backward (working memory), TMT-A and B (processing speed and executive functioning, respectively), phonemic and category fluency (language), and the Hopkins Verbal Learning Test-revised (memory encoding, recall, and recognition)	Relatively high frequency of cognitive impairment several months after COVID-19 recovery. Deficits in executive functioning, processing speed, category fluency, memory encoding, and recall were predominant among hospitalized patients.	Good
Davis et al., 2021 [26]	Two surveys with platform Qualtrics (257 questions) + MRI if memory and/or cognitive dysfunction symptoms	88.0% of the participants experienced cognitive dysfunction and/or memory loss. By 7 months, lots of the respondents have not yet recovered and have not returned to previous levels of work, and still experience significant symptom burden.	Good
Del Brutto et al., 2021 [27]	MoCA	Cognitive decline was highlighted in patients with mild COVID-19 infection	Good
Dressing et al., 2021 [28]	Neuropsychological and psychiatric evaluations and Cerebral 18F-FDG PET imaging on 14/31 patients, Hopkins Verbal Learning Test-Revised, Brief Visuospatial Memory Test-Revised (BVMT-R), DST, TMT-A and B, Color-Word Interference Test (FWIT), Symbol-Digit Modalities Test (SDMT), semantic and letter fluency test	Minor deficits in cognitive testing six months after infection, suggesting that neuronal causes could possibly be related to the high prevalence of tiredness.	Good
Hampshire et al., 2021 [29]	Great British intelligence Test	Recovered COVID-19 patients exhibited significant cognitive deficits vs. controls. Impairments were higher for people who had been hospitalized, but also for non-hospitalized cases who had biological confirmation of COVID-19 infection.	Good
Hosp et al., 2021 [30]	The German version of the MoCA and MRI, FDG-PET-SCAN, CSF analysis	MoCA performance was impaired in 18/26 patients. 18FDG PET revealed pathological results in 10/15 patients with predominant frontoparietal hypometabolism.	Good
Lamontagne et al., 2021 [31]	Self-reported measures of stress, depression, and anhedonia, as well as the Attention Network Test and cognitive abilities (Attentional Control Scale)	Selective impairment in attention was observed in the COVID-19 group, marked by deficits in executive functioning while alerting and orienting abilities remained intact. Effects were most pronounced among individuals diagnosed 1–4 months before assessment. The COVID-19 recovered group scored significantly higher on perceived stress.	Good
Mattioli et al., 2021 [32]	Controlled Oral Word Association by categories, California Verbal Learning Test, TEA attention test, visual reaction times, auditory reaction times, number of errors and of omissions for attention Tower of London test, and MMSE.	No neurological deficits or cognitive impairment in mild-moderate COVID-19 patients 4 months after the diagnosis, but severe emotional disorders were confirmed.	Good
Méndez et al., 2021 [33]	Phone questionnaire	Declined cognitive function, psychiatric morbidity and low QoL are observable in moderate to severe COVID-19 survivors, 1 year after hospital discharge.	Good
Miskowiak et al., 2021 [34]	Cognitive failure questionnaires and performance-based cognition test battery (Screen for Cognitive Impairment in Psychiatry Danish version and TMT-B)	59–65% of the 29 patients experience cognitive impairments 3–4 months after hospitalization. More than 80% of patients reported severe daily cognitive difficulties. Poorer pulmonary function and more respiratory symptoms after recovery were associated with more cognitive impairments, suggesting a potential link with brain hypoxia.	Good
Norrefalk et al., 2021 [35]	Questionnaire (Functional Compass COVID-19)	Persistent fatigue seems to be the most annoying symptom of post-COVID syndromes in mildly infected participants who developed pronounced impairments in functioning and disability.	Fair
Patel et al., 2021 [36]	MoCA	Cognitive improvement over time may reflect natural recovery and/or rehabilitation intervention effects	Fair
Poletti et al., 2021 [37]	Neuropsychological and psychiatric evaluations	Cognitive impairment in at least one cognitive function was observed in 1-,3-, and 6-month follow-up patients with no significant difference in cognitive performances between 1-,3-, and 6 months. COVID-19 patients performed the same as healthy control in working memory and verbal memory. Depressive psychopathology was the most predominant factor which, in turn, interacts with cognitive functions in determining the quality of life. Sequelae include signs of cognitive impairment, persist up to 6 months after hospital discharge, and affect the quality of life.	Good
Rousseau et al., 2021 [38]	MoCA	The burden of severe COVID-19 and prolonged ICU stay was considerable after 3 months, affecting both functional status and biological parameters.	Good
Solaro et al., 2021 [39]	MoCA	A significant cognitive impairment was observed in young sub-acute COVID-19 subjects at the time of hospital discharge.	Fair
Van den Borst et al., 2021 [40]	Questionnaires on mental, cognitive, health status, and QoL	Severe problems in several health domains were observed in a substantial number of COVID-19 patients.	Good
Vyas et al., 2021 [41]	Brain fog symptoms questionnaire (with a validated measure)	Brain fog was frequent in COVID-19 survivors and significantly higher with COVID-19 severity and in patients who received oxygen or who were placed under ventilator	Good
Zhou et al., 2021 [42]	Association analysis across 974 phenotypes and 30 blood biomarkers	Pre-existing Alzheimer’s disease and dementia were identified as top risk factors for hospital admission due to COVID-19, highlighting the necessity of providing adequate protective care for patients with cognitive disorders with this infection.	Good
Aiello et al., 2022 [43]	MoCA and MMSE	MMSE and MoCA are able to detect sequelae deficits in COVID-19-recovered individuals who were or were not at risk for cognitive deficits	Good
Bonizzato et al., 2022 [44]	MoCA and MMSE	Significant amelioration was found in neuropsychiatry inventory scores, a qualitative improvement has been detected at all tests, after discharge, and after 3 months.	Fair
Del Brutto et al., 2022 [45]	MoCA	Long COVID-related cognitive decline may spontaneously improve over time.	Good
Liu et al., 2022 [46]	Phone questionnaire (Telephone Interview of Cognitive Status-40 (TICS-40) and Informant Questionnaire on Cognitive Decline in the Elderly (IQCODE))	COVID-19 survival was associated with an increase in the risk of longitudinal cognitive decline	Good
Tabacof et al., 2022 [47]	RedCap Survey (Neuro-Qol, EQ-5D-5L)	Persistent symptoms associated with post-acute COVID-19 syndrome seem to impact physical and cognitive function, health-related quality of life, and participation in society.	Fair

BVMT-R: Brief Visuospatial Memory Test-Revised. CPT: Continuous Performance Test. DST: Digital Span Test. FWIT: Color-Word Interference Test/IQCODE: Informant Questionnaire on Cognitive Decline in the Elderly. MMSE: Mini-Mental State Examination, MoCA: Montreal Cognitive Assessment. QoL: Quality of Life. SDMT: Symbol-Digit Modalities Test. SCT: Sign Coding Test. TICS-40: Telephone Interview of Cognitive Status-40. TMT: Trail Making Test. * Quality of the study was done with the NOS, the scores were then transformed to AHQR standards.

## Data Availability

Not applicable.

## References

[1] Dong E., Du H., Gardner L. (2020). An Interactive Web-Based Dashboard to Track COVID-19 in Real Time. Lancet Infect. Dis..

[2] Gabutti G., d’Anchera E., Sandri F., Savio M., Stefanati A. (2020). Coronavirus: Update Related to the Current Outbreak of COVID-19. Infect. Dis. Ther..

[3] 2019 Novel Coronavirus COVID-19 (2019-NCoV) Data Repository by Johns Hopkins CSSE. https://github.com/CSSEGISandData/COVID-19.

[4] Wu Z., McGoogan J.M. (2020). Characteristics of and Important Lessons from the Coronavirus Disease 2019 (COVID-19) Outbreak in China: Summary of a Report of 72 314 Cases from the Chinese Center for Disease Control and Prevention. JAMA.

[5] Burn E., Tebé C., Fernandez-Bertolin S., Aragon M., Recalde M., Roel E., Prats-Uribe A., Prieto-Alhambra D., Duarte-Salles T. (2021). The Natural History of Symptomatic COVID-19 during the First Wave in Catalonia. Nat. Commun..

[6] Higgins V., Sohaei D., Diamandis E.P., Prassas I. (2021). COVID-19: From an Acute to Chronic Disease? Potential Long-Term Health Consequences. Crit. Rev. Clin. Lab. Sci..

[7] Soriano J.B., Murthy S., Marshall J.C., Relan P., Diaz J.V., WHO Clinical Case Definition Working Group on Post-COVID-19 Condition (2022). A Clinical Case Definition of Post-COVID-19 Condition by a Delphi Consensus. Lancet Infect. Dis..

[8] Callard F., Perego E. (2021). How and Why Patients Made Long Covid. Soc. Sci. Med..

[9] Vogel A., Salem L.C., Andersen B.B., Waldemar G. (2016). Differences in Quantitative Methods for Measuring Subjective Cognitive Decline—Results from a Prospective Memory Clinic Study. Int. Psychogeriatr..

[10] Ofen N., Shing Y.L. (2013). From Perception to Memory: Changes in Memory Systems across the Lifespan. Neurosci. Biobehav. Rev..

[11] Bonnechère B. (2022). Evaluation of Processing Speed of Different Cognitive Functions Across the Life Span Using Cognitive Mobile Games. Games Health J..

[12] Staffolani S., Iencinella V., Cimatti M., Tavio M. (2022). Long COVID-19 Syndrome as a Fourth Phase of SARS-CoV-2 Infection. Infez. Med..

[13] Methley A.M., Campbell S., Chew-Graham C., McNally R., Cheraghi-Sohi S. (2014). PICO, PICOS and SPIDER: A Comparison Study of Specificity and Sensitivity in Three Search Tools for Qualitative Systematic Reviews. BMC Health Serv. Res..

[14] Lo C.K.-L., Mertz D., Loeb M. (2014). Newcastle-Ottawa Scale: Comparing Reviewers’ to Authors’ Assessments. BMC Med. Res. Methodol..

[15] Shamsrizi P., Gladstone B.P., Carrara E., Luise D., Cona A., Bovo C., Tacconelli E. (2020). Variation of Effect Estimates in the Analysis of Mortality and Length of Hospital Stay in Patients with Infections Caused by Bacteria-Producing Extended-Spectrum Beta-Lactamases: A Systematic Review and Meta-Analysis. BMJ Open.

[16] Higgins J.P.T., Thomas J., Chandler J., Cumpston M., Li T., Page M., Welch V. (2019). Cochrane Handbook for Systematic Reviews of Interventions.

[17] Carter E.C., Schönbrodt F.D., Gervais W.M., Hilgard J. (2019). Correcting for Bias in Psychology: A Comparison of Meta-Analytic Methods. Adv. Methods Pract. Psychol. Sci..

[18] Sterne J.A.C., Sutton A.J., Ioannidis J.P.A., Terrin N., Jones D.R., Lau J., Carpenter J., Rücker G., Harbord R.M., Schmid C.H. (2011). Recommendations for Examining and Interpreting Funnel Plot Asymmetry in Meta-Analyses of Randomised Controlled Trials. BMJ.

[19] Pustejovsky J.E., Rodgers M.A. (2019). Testing for Funnel Plot Asymmetry of Standardized Mean Differences. Res. Synth. Methods.

[20] Liberati A., Altman D.G., Tetzlaff J., Mulrow C., Gøtzsche P.C., Ioannidis J.P.A., Clarke M., Devereaux P.J., Kleijnen J., Moher D. (2009). The PRISMA Statement for Reporting Systematic Reviews and Meta-Analyses of Studies That Evaluate Health Care Interventions: Explanation and Elaboration. PLoS Med..

[21] Woo M.S., Malsy J., Pöttgen J., Seddiq Zai S., Ufer F., Hadjilaou A., Schmiedel S., Addo M.M., Gerloff C., Heesen C. (2020). Frequent Neurocognitive Deficits after Recovery from Mild COVID-19. Brain Commun..

[22] Zhou H., Lu S., Chen J., Wei N., Wang D., Lyu H., Shi C., Hu S. (2020). The Landscape of Cognitive Function in Recovered COVID-19 Patients. J. Psychiatr. Res..

[23] Alemanno F., Houdayer E., Parma A., Spina A., Del Forno A., Scatolini A., Angelone S., Brugliera L., Tettamanti A., Beretta L. (2021). COVID-19 Cognitive Deficits after Respiratory Assistance in the Subacute Phase: A COVID-Rehabilitation Unit Experience. PLoS ONE.

[24] Amalakanti S., Arepalli K.V.R., Jillella J.P. (2021). Cognitive Assessment in Asymptomatic COVID-19 Subjects. Virusdisease.

[25] Becker J.H., Lin J.J., Doernberg M., Stone K., Navis A., Festa J.R., Wisnivesky J.P. (2021). Assessment of Cognitive Function in Patients After COVID-19 Infection. JAMA Netw. Open.

[26] Davis H.E., Assaf G.S., McCorkell L., Wei H., Low R.J., Re’em Y., Redfield S., Austin J.P., Akrami A. (2021). Characterizing Long COVID in an International Cohort: 7 Months of Symptoms and Their Impact. EClinicalMedicine.

[27] Del Brutto O.H., Wu S., Mera R.M., Costa A.F., Recalde B.Y., Issa N.P. (2021). Cognitive Decline among Individuals with History of Mild Symptomatic SARS-CoV-2 Infection: A Longitudinal Prospective Study Nested to a Population Cohort. Eur. J. Neurol..

[28] Dressing A., Bormann T., Blazhenets G., Schroeter N., Walter L.I., Thurow J., August D., Hilger H., Stete K., Gerstacker K. (2021). Neuropsychological Profiles and Cerebral Glucose Metabolism in Neurocognitive Long COVID-Syndrome. J. Nucl. Med..

[29] Hampshire A., Trender W., Chamberlain S.R., Jolly A.E., Grant J.E., Patrick F., Mazibuko N., Williams S.C., Barnby J.M., Hellyer P. (2021). Cognitive Deficits in People Who Have Recovered from COVID-19. EClinicalMedicine.

[30] Hosp J.A., Dressing A., Blazhenets G., Bormann T., Rau A., Schwabenland M., Thurow J., Wagner D., Waller C., Niesen W.D. (2021). Cognitive Impairment and Altered Cerebral Glucose Metabolism in the Subacute Stage of COVID-19. Brain.

[31] Lamontagne S.J., Winters M.F., Pizzagalli D.A., Olmstead M.C. (2021). Post-Acute Sequelae of COVID-19: Evidence of Mood & Cognitive Impairment. Brain Behav. Immun. Health.

[32] Mattioli F., Stampatori C., Righetti F., Sala E., Tomasi C., De Palma G. (2021). Neurological and Cognitive Sequelae of Covid-19: A Four Month Follow-Up. J. Neurol..

[33] Méndez R., Balanzá-Martínez V., Luperdi S.C., Estrada I., Latorre A., González-Jiménez P., Feced L., Bouzas L., Yépez K., Ferrando A. (2021). Short-Term Neuropsychiatric Outcomes and Quality of Life in COVID-19 Survivors. J. Intern. Med..

[34] Miskowiak K.W., Johnsen S., Sattler S.M., Nielsen S., Kunalan K., Rungby J., Lapperre T., Porsberg C.M. (2021). Cognitive Impairments Four Months after COVID-19 Hospital Discharge: Pattern, Severity and Association with Illness Variables. Eur. Neuropsychopharmacol..

[35] Norrefalk J.-R., Borg K., Bileviciute-Ljungar I. (2021). Self-Scored Impairments in Functioning and Disability in Post-COVID Syndrome Following Mild COVID-19 Infection. J. Rehabil. Med..

[36] Patel R., Savrides I., Cahalan C., Doulatani G., O’Dell M.W., Toglia J., Jaywant A. (2021). Cognitive Impairment and Functional Change in COVID-19 Patients Undergoing Inpatient Rehabilitation. Int. J. Rehabil. Res..

[37] Poletti S., Palladini M., Mazza M.G., De Lorenzo R. (2021). COVID-19 BioB Outpatient Clinic Study Group; Furlan, R.; Ciceri, F.; Rovere-Querini, P.; Benedetti, F. Long-Term Consequences of COVID-19 on Cognitive Functioning up to 6 Months after Discharge: Role of Depression and Impact on Quality of Life. Eur. Arch. Psychiatry Clin. Neurosci..

[38] Rousseau A.-F., Minguet P., Colson C., Kellens I., Chaabane S., Delanaye P., Cavalier E., Chase J.G., Lambermont B., Misset B. (2021). Post-Intensive Care Syndrome after a Critical COVID-19: Cohort Study from a Belgian Follow-up Clinic. Ann. Intensive Care.

[39] Solaro C., Gamberini G., Masuccio F.G. (2021). Cognitive Impairment in Young COVID-19 Patients: The Tip of the Iceberg?. Neurol. Sci..

[40] van den Borst B., Peters J.B., Brink M., Schoon Y., Bleeker-Rovers C.P., Schers H., van Hees H.W.H., van Helvoort H., van den Boogaard M., van der Hoeven H. (2021). Comprehensive Health Assessment 3 Months After Recovery from Acute Coronavirus Disease 2019 (COVID-19). Clin. Infect. Dis..

[41] Vyas A., Raja Panwar V., Mathur V., Patel P., Mathur S., Sharma A., Babu Panwar R., Gupta R. (2021). Mild Cognitive Impairment in COVID-19 Survivors: Measuring the Brain Fog. Int. J. Ment. Health.

[42] Zhou J., Liu C., Sun Y., Huang W., Ye K. (2021). Cognitive Disorders Associated with Hospitalization of COVID-19: Results from an Observational Cohort Study. Brain Behav. Immun..

[43] Aiello E.N., Fiabane E., Manera M.R., Radici A., Grossi F., Ottonello M., Pain D., Pistarini C. (2022). Screening for Cognitive Sequelae of SARS-CoV-2 Infection: A Comparison between the Mini-Mental State Examination (MMSE) and the Montreal Cognitive Assessment (MoCA). Neurol. Sci..

[44] Bonizzato S., Ghiggia A., Ferraro F., Galante E. (2022). Cognitive, Behavioral, and Psychological Manifestations of COVID-19 in Post-Acute Rehabilitation Setting: Preliminary Data of an Observational Study. Neurol. Sci..

[45] Del Brutto O.H., Rumbea D.A., Recalde B.Y., Mera R.M. (2022). Cognitive Sequelae of Long COVID May Not Be Permanent: A Prospective Study. Eur. J. Neurol..

[46] Liu Y.-H., Chen Y., Wang Q.-H., Wang L.-R., Jiang L., Yang Y., Chen X., Li Y., Cen Y., Xu C. (2022). One-Year Trajectory of Cognitive Changes in Older Survivors of COVID-19 in Wuhan, China: A Longitudinal Cohort Study. JAMA Neurol..

[47] Tabacof L., Tosto-Mancuso J., Wood J., Cortes M., Kontorovich A., McCarthy D., Rizk D., Rozanski G., Breyman E., Nasr L. (2022). Post-Acute COVID-19 Syndrome Negatively Impacts Physical Function, Cognitive Function, Health-Related Quality of Life, and Participation. Am. J. Phys. Med. Rehabil..

[48] Nasreddine Z.S., Phillips N.A., Bédirian V., Charbonneau S., Whitehead V., Collin I., Cummings J.L., Chertkow H. (2005). The Montreal Cognitive Assessment, MoCA: A Brief Screening Tool for Mild Cognitive Impairment. J. Am. Geriatr. Soc..

[49] Lou J.J., Movassaghi M., Gordy D., Olson M.G., Zhang T., Khurana M.S., Chen Z., Perez-Rosendahl M., Thammachantha S., Singer E.J. (2021). Neuropathology of COVID-19 (Neuro-COVID): Clinicopathological Update. Free Neuropathol..

[50] Al-Aly Z., Bowe B., Xie Y. (2022). Long COVID after Breakthrough SARS-CoV-2 Infection. Nat. Med..

[51] Ayoubkhani D., Bermingham C., Pouwels K.B., Glickman M., Nafilyan V., Zaccardi F., Khunti K., Alwan N.A., Walker A.S. (2022). Trajectory of Long Covid Symptoms after COVID-19 Vaccination: Community Based Cohort Study. BMJ.

[52] Antonelli M., Penfold R.S., Merino J., Sudre C.H., Molteni E., Berry S., Canas L.S., Graham M.S., Klaser K., Modat M. (2022). Risk Factors and Disease Profile of Post-Vaccination SARS-CoV-2 Infection in UK Users of the COVID Symptom Study App: A Prospective, Community-Based, Nested, Case-Control Study. Lancet Infect. Dis..

[53] CDC VAERS COVID Vaccine Adverse Event Reports. https://openvaers.com/covid-data.

[54] Asadi-Pooya A.A., Nemati H., Shahisavandi M., Akbari A., Emami A., Lotfi M., Rostamihosseinkhani M., Barzegar Z., Kabiri M., Zeraatpisheh Z. (2021). Long COVID in Children and Adolescents. World J. Pediatr..

[55] Radtke T., Ulyte A., Puhan M.A., Kriemler S. (2021). Long-Term Symptoms After SARS-CoV-2 Infection in Children and Adolescents. JAMA.

[56] Stephenson T., Allin B., Nugawela M.D., Rojas N., Dalrymple E., Pinto Pereira S., Soni M., Knight M., Cheung E.Y., Heyman I. (2022). Long COVID (Post-COVID-19 Condition) in Children: A Modified Delphi Process. Arch. Dis. Child..

[57] Del Valle D.M., Kim-Schulze S., Huang H.-H., Beckmann N.D., Nirenberg S., Wang B., Lavin Y., Swartz T.H., Madduri D., Stock A. (2020). An Inflammatory Cytokine Signature Predicts COVID-19 Severity and Survival. Nat. Med..

[58] Osimo E.F., Baxter L.J., Lewis G., Jones P.B., Khandaker G.M. (2019). Prevalence of Low-Grade Inflammation in Depression: A Systematic Review and Meta-Analysis of CRP Levels. Psychol. Med..

[59] Alonso-Lana S., Marquié M., Ruiz A., Boada M. (2020). Cognitive and Neuropsychiatric Manifestations of COVID-19 and Effects on Elderly Individuals with Dementia. Front. Aging Neurosci..

[60] Menard C., Pfau M.L., Hodes G.E., Kana V., Wang V.X., Bouchard S., Takahashi A., Flanigan M.E., Aleyasin H., LeClair K.B. (2017). Social Stress Induces Neurovascular Pathology Promoting Depression. Nat. Neurosci..

[61] Iadecola C., Anrather J., Kamel H. (2020). Effects of COVID-19 on the Nervous System. Cell.

[62] Hoffmann M., Kleine-Weber H., Schroeder S., Krüger N., Herrler T., Erichsen S., Schiergens T.S., Herrler G., Wu N.-H., Nitsche A. (2020). SARS-CoV-2 Cell Entry Depends on ACE2 and TMPRSS2 and Is Blocked by a Clinically Proven Protease Inhibitor. Cell.

[63] De Roeck A., Van Broeckhoven C., Sleegers K. (2019). The Role of ABCA7 in Alzheimer’s Disease: Evidence from Genomics, Transcriptomics and Methylomics. Acta Neuropathol..

[64] Zeng N., Zhao Y.-M., Yan W., Li C., Lu Q.-D., Liu L., Ni S.-Y., Mei H., Yuan K., Shi L. (2022). A Systematic Review and Meta-Analysis of Long Term Physical and Mental Sequelae of COVID-19 Pandemic: Call for Research Priority and Action. Mol. Psychiatry.

[65] Bronheim R.S., Cotter E., Skolasky R.L. (2022). Cognitive Impairment Is Associated with Greater Preoperative Symptoms, Worse Health-Related Quality of Life, and Reduced Likelihood of Recovery after Cervical and Lumbar Spine Surgery. N. Am. Spine Soc. J..

[66] Ma Y., Deng J., Liu Q., Du M., Liu M., Liu J. (2022). Long-Term Consequences of COVID-19 at 6 Months and Above: A Systematic Review and Meta-Analysis. Int. J. Environ. Res. Public Health.

[67] Pistarini C., Fiabane E., Houdayer E., Vassallo C., Manera M.R., Alemanno F. (2021). Cognitive and Emotional Disturbances Due to COVID-19: An Exploratory Study in the Rehabilitation Setting. Front. Neurol..

[68] Fugazzaro S., Contri A., Esseroukh O., Kaleci S., Croci S., Massari M., Facciolongo N.C., Besutti G., Iori M., Salvarani C. (2022). Rehabilitation Interventions for Post-Acute COVID-19 Syndrome: A Systematic Review. Int. J. Environ. Res. Public Health.

[69] Vance H., Maslach A., Stoneman E., Harmes K., Ransom A., Seagly K., Furst W. (2021). Addressing Post-COVID Symptoms: A Guide for Primary Care Physicians. J. Am. Board Fam. Med..

[70] Dixit S., Borghi-Silva A., Bairapareddy K.C. (2021). Revisiting Pulmonary Rehabilitation during COVID-19 Pandemic: A Narrative Review. Rev. Cardiovasc. Med..

[71] Halle M., Bloch W., Niess A.M., Predel H.-G., Reinsberger C., Scharhag J., Steinacker J., Wolfarth B., Scherr J., Niebauer J. (2021). Exercise and Sports after COVID-19—Guidance from a Clinical Perspective. Transl. Sports Med..

[72] Jimeno-Almazán A., Pallarés J.G., Buendía-Romero Á., Martínez-Cava A., Franco-López F., Sánchez-Alcaraz Martínez B.J., Bernal-Morel E., Courel-Ibáñez J. (2021). Post-COVID-19 Syndrome and the Potential Benefits of Exercise. Int. J. Environ. Res. Public Health.

[73] Shaw T., McGregor D., Brunner M., Keep M., Janssen A., Barnet S. (2017). What Is EHealth (6)? Development of a Conceptual Model for EHealth: Qualitative Study with Key Informants. J. Med. Internet Res..

[74] Cottrell M.A., Galea O.A., O’Leary S.P., Hill A.J., Russell T.G. (2017). Real-Time Telerehabilitation for the Treatment of Musculoskeletal Conditions Is Effective and Comparable to Standard Practice: A Systematic Review and Meta-Analysis. Clin. Rehabil..

[75] Howard I.M., Kaufman M.S. (2018). Telehealth Applications for Outpatients with Neuromuscular or Musculoskeletal Disorders. Muscle Nerve.

[76] Gonzalez-Gerez J.J., Saavedra-Hernandez M., Anarte-Lazo E., Bernal-Utrera C., Perez-Ale M., Rodriguez-Blanco C. (2021). Short-Term Effects of a Respiratory Telerehabilitation Program in Confined COVID-19 Patients in the Acute Phase: A Pilot Study. Int. J. Environ. Res. Public Health.

[77] Li J., Xia W., Zhan C., Liu S., Yin Z., Wang J., Chong Y., Zheng C., Fang X., Cheng W. (2021). A Telerehabilitation Programme in Post-Discharge COVID-19 Patients (TERECO): A Randomised Controlled Trial. Thorax.

[78] Rodríguez-Blanco C., Bernal-Utrera C., Anarte-Lazo E., Saavedra-Hernandez M., De-La-Barrera-Aranda E., Serrera-Figallo M.A., Gonzalez-Martin M., Gonzalez-Gerez J.J. (2022). Breathing Exercises versus Strength Exercises through Telerehabilitation in Coronavirus Disease 2019 Patients in the Acute Phase: A Randomized Controlled Trial. Clin. Rehabil..

[79] Livingston G., Huntley J., Sommerlad A., Ames D., Ballard C., Banerjee S., Brayne C., Burns A., Cohen-Mansfield J., Cooper C. (2020). Dementia Prevention, Intervention, and Care: 2020 Report of the Lancet Commission. Lancet.

[80] Sujkowski A., Hong L., Wessells R.J., Todi S.V. (2022). The Protective Role of Exercise against Age-Related Neurodegeneration. Ageing Res. Rev..

[81] Jia R.-X., Liang J.-H., Xu Y., Wang Y.-Q. (2019). Effects of Physical Activity and Exercise on the Cognitive Function of Patients with Alzheimer Disease: A Meta-Analysis. BMC Geriatr..

[82] Panza G.A., Taylor B.A., MacDonald H.V., Johnson B.T., Zaleski A.L., Livingston J., Thompson P.D., Pescatello L.S. (2018). Can Exercise Improve Cognitive Symptoms of Alzheimer’s Disease?. J. Am. Geriatr. Soc..

[83] Brasure M., Desai P., Davila H., Nelson V.A., Calvert C., Jutkowitz E., Butler M., Fink H.A., Ratner E., Hemmy L.S. (2018). Physical Activity Interventions in Preventing Cognitive Decline and Alzheimer-Type Dementia: A Systematic Review. Ann. Intern. Med..

[84] Meng Q., Yin H., Wang S., Shang B., Meng X., Yan M., Li G., Chu J., Chen L. (2022). The Effect of Combined Cognitive Intervention and Physical Exercise on Cognitive Function in Older Adults with Mild Cognitive Impairment: A Meta-Analysis of Randomized Controlled Trials. Aging Clin. Exp. Res..

[85] Wahezi S.E., Kohan L.R., Spektor B., Brancolini S., Emerick T., Fronterhouse J.M., Luedi M.M., Colon M.A., Kitei P.M., Anitescu M. (2021). Telemedicine and Current Clinical Practice Trends in the COVID-19 Pandemic. Best Pract. Res. Clin. Anaesthesiol..

[86] Van Hove O., Gillet A., Tack J., Reychler G., Guatteri M., Ballarin A., Thomas J., Espinoza R., Bonnier F., Norrenberg M. (2022). Development of a Medium Care Unit Using an Inexperienced Respiratory Staff: Lessons Learned during the COVID-19 Pandemic. Int. J. Environ. Res. Public Health.

[87] Lugo-Agudelo L.H., Cruz Sarmiento K.M., Spir Brunal M.A., Velásquez Correa J.C., Posada Borrero A.M., Fernanda Mesa Franco L., Di Dio Castagna Ianini R., Ramírez Pérez Lis P.A., Vélez C.M., Patiño Lugo D.F. (2021). Adaptations for Rehabilitation Services during the COVID-19 Pandemic Proposed by Scientific Organizations and Rehabilitation Professionals. J. Rehabil. Med..

[88] Bennell K.L., Marshall C.J., Dobson F., Kasza J., Lonsdale C., Hinman R.S. (2019). Does a Web-Based Exercise Programming System Improve Home Exercise Adherence for People With Musculoskeletal Conditions?: A Randomized Controlled Trial. Am. J. Phys. Med. Rehabil..

[89] Lambert T.E., Harvey L.A., Avdalis C., Chen L.W., Jeyalingam S., Pratt C.A., Tatum H.J., Bowden J.L., Lucas B.R. (2017). An App with Remote Support Achieves Better Adherence to Home Exercise Programs than Paper Handouts in People with Musculoskeletal Conditions: A Randomised Trial. J. Physiother..

[90] Lawford B.J., Delany C., Bennell K.L., Hinman R.S. (2018). “I Was Really Sceptical...But It Worked Really Well”: A Qualitative Study of Patient Perceptions of Telephone-Delivered Exercise Therapy by Physiotherapists for People with Knee Osteoarthritis. Osteoarthr. Cartil..

[91] Moffet H., Tousignant M., Nadeau S., Mérette C., Boissy P., Corriveau H., Marquis F., Cabana F., Belzile É.L., Ranger P. (2017). Patient Satisfaction with In-Home Telerehabilitation After Total Knee Arthroplasty: Results from a Randomized Controlled Trial. Telemed. J. e-Health.

[92] Bonnechère B., Langley C., Sahakian B.J. (2020). The Use of Commercial Computerised Cognitive Games in Older Adults: A Meta-Analysis. Sci. Rep..

[93] Zhang H., Huntley J., Bhome R., Holmes B., Cahill J., Gould R.L., Wang H., Yu X., Howard R. (2019). Effect of Computerised Cognitive Training on Cognitive Outcomes in Mild Cognitive Impairment: A Systematic Review and Meta-Analysis. BMJ Open.

[94] Ye M., Zhao B., Liu Z., Weng Y., Zhou L. (2020). Effectiveness of Computer-Based Training on Post-Stroke Cognitive Rehabilitation: A Systematic Review and Meta-Analysis. Neuropsychol. Rehabil..

[95] Orgeta V., McDonald K.R., Poliakoff E., Hindle J.V., Clare L., Leroi I. (2020). Cognitive Training Interventions for Dementia and Mild Cognitive Impairment in Parkinson’s Disease. Cochrane Database Syst. Rev..

[96] Lampit A., Heine J., Finke C., Barnett M.H., Valenzuela M., Wolf A., Leung I.H.K., Hill N.T.M. (2019). Computerized Cognitive Training in Multiple Sclerosis: A Systematic Review and Meta-Analysis. Neurorehabilit. Neural Repair.

[97] Dixit S., Nandakumar G. (2021). Promoting Healthy Lifestyles Using Information Technology during the COVID-19 Pandemic. Rev. Cardiovasc. Med..

[98] Marra C., Gordon W.J., Stern A.D. (2021). Use of Connected Digital Products in Clinical Research Following the COVID-19 Pandemic: A Comprehensive Analysis of Clinical Trials. BMJ Open.

[99] Carl J.R., Jones D.J., Lindhiem O.J., Doss B.D., Weingardt K.R., Timmons A.C., Comer J.S. (2021). Regulating Digital Therapeutics for Mental Health: Opportunities, Challenges, and the Essential Role of Psychologists. Br. J. Clin. Psychol..

[100] Scott Kruse C., Karem P., Shifflett K., Vegi L., Ravi K., Brooks M. (2018). Evaluating Barriers to Adopting Telemedicine Worldwide: A Systematic Review. J. Telemed. Telecare.

[101] Rangachari P., Mushiana S.S., Herbert K. (2021). A Narrative Review of Factors Historically Influencing Telehealth Use across Six Medical Specialties in the United States. Int. J. Environ. Res. Public Health.

[102] Almathami H.K.Y., Win K.T., Vlahu-Gjorgievska E. (2020). Barriers and Facilitators That Influence Telemedicine-Based, Real-Time, Online Consultation at Patients’ Homes: Systematic Literature Review. J. Med. Internet Res..

[103] Engelsma T., Jaspers M.W.M., Peute L.W. (2021). Considerate MHealth Design for Older Adults with Alzheimer’s Disease and Related Dementias (ADRD): A Scoping Review on Usability Barriers and Design Suggestions. Int. J. Med. Inf..

